# C2-Related Radicular Pain Due to a Posterior Arch Defect of C1 in an Elderly Patient: A Case Report

**DOI:** 10.7759/cureus.96039

**Published:** 2025-11-03

**Authors:** Yasushi Murakami, Yasushi Fujiwara, Ryo Ota, Shinji Kotaka, Nobuo Adachi

**Affiliations:** 1 Orthopaedic and Microscopic Spine and Spinal Cord Surgery Center, Hiroshima City North Medical Center Asa Citizens Hospital, Hiroshima, JPN; 2 Orthopedic Surgery, Graduate School of Biomedical and Health Sciences, Hiroshima University, Hiroshima, JPN

**Keywords:** c1 posterior arch defect, c2 radiculopathy, elderly, microscopic decompression, occipital neuralgia, rheumatoid arthritis

## Abstract

A 94-year-old woman with rheumatoid arthritis presented with sudden-onset, intractable right occipital pain without trauma. Radiological studies revealed C1-C2 foraminal stenosis and spinal cord compression caused by an unrecognized posterior arch defect of C1. Conservative management failed, and microscopic decompression without fusion was performed. Intraoperatively, a small bone fragment was found compressing the right C2 nerve root and was carefully removed. Her visual analog scale (VAS) pain score improved from 100 mm preoperatively to 50 mm immediately after surgery and to 5 mm at the two-year follow-up. This case highlights the importance of considering C1-C2 foraminal pathology and subtle C1 posterior arch defects as potential causes of occipital neuralgia or C2 radiculopathy in elderly patients with rheumatoid arthritis. Early recognition and minimally invasive decompression can achieve favorable outcomes even without fusion.

## Introduction

C2 radiculopathy is a recognized cause of severe occipital neuralgia. Various mechanisms have been described, including mechanical compression from spondylosis [1-5], venous plexus enlargement [6-8], vertebral artery impingement [9], and hypertrophy of the atlantoepistrophic ligament [10].

Among these, spondylosis is the most frequently encountered cause. Fujiwara et al. [11] reported that unilateral spondylotic changes can lead to foraminal stenosis at the C1-C2 level, resulting in C2 radiculopathy, which was successfully treated using microscopic posterior foraminotomy.

In elderly patients with rheumatoid arthritis (RA), structural changes such as atlantoaxial subluxation, facet arthrosis, and posterior arch erosion may coexist, making diagnosis complex.

We report an elderly woman with RA who developed C2-related radicular pain due to a posterior arch defect of C1. Although the lesion was not clearly identifiable on preoperative imaging, intraoperative microscopic decompression achieved significant pain relief without fusion. In this report, we describe the case and discuss the diagnostic and surgical considerations.

## Case presentation

A 94-year-old woman had experienced mild numbness in all four limbs for several years due to atlantoaxial subluxation and cervical spinal canal stenosis associated with rheumatoid arthritis. Surgical treatment had not been pursued because of the patient’s comorbidities, including rheumatoid arthritis-related fragility and osteoporosis. One week prior to admission, she developed sudden-onset, intractable right occipital neuralgia and numbness, without any history of trauma. She presented to our emergency department.

The patient had been treated for rheumatoid arthritis and osteoporosis at another hospital. Her medications included subcutaneous abatacept (125 mg weekly) and oral methotrexate (2 mg daily). Rheumatoid arthritis had been managed since age 60 and was clinically stable without active synovitis at presentation. For osteoporosis, she had been taking oral minodronic acid hydrate (Bonoteo 50 mg monthly) and alfacalcidol (0.5 µg daily). The most recent Clinical Disease Activity Index (CDAI)/Disease Activity Score (DAS) and dual-energy X-ray absorptiometry (DEXA) results were unavailable because they were followed externally. She sustained a left humeral fracture at age 76 after a minor fall.

**Figure 1:**
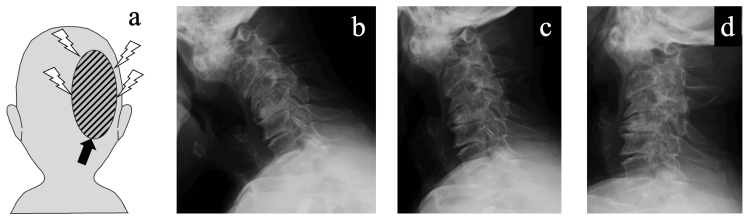
Pain distribution and dynamic radiographic findings. (a) Schematic illustration showing the patient’s right occipital neuralgia localized to the greater occipital nerve territory. Black arrows indicate pain radiation. (b–d) Dynamic lateral cervical radiographs showing atlantoaxial subluxation and cervical kyphosis. In the neutral position (c), the atlantodental interval (ADI) was 4.0 mm and the space available for the cord (SAC) was 10.8 mm. Only minimal changes in ADI and SAC were observed in flexion (b) and extension (d), suggesting no significant dynamic instability.

The pain and numbness were localized to the distribution of the greater occipital nerve. Her visual analog scale (VAS) score for pain was 100 mm. The pain radiated from the upper cervical spine to the right occiput (Figure 1A) and was aggravated by neck movements, particularly left lateral flexion, right rotation, and extension. She reported no upper extremity pain or associated symptoms such as dizziness or autonomic disturbances.

Plain radiographs showed advanced spondlotic changes, kyphotic alignment, and atlantoaxial subluxation (Figure 1B-1D). In the neutral position, the atlantodental interval (ADI) measured 4.0 mm and the space available for the cord (SAC) was 10.8 mm (Figure 1C). In flexion, the ADI increased to 4.7 mm and the SAC decreased to 8.7 mm (Figure 1B). In extension, the ADI decreased to 3.0 mm, with the SAC remaining at 8.7 mm (Figure 1D).

**Figure 2:**
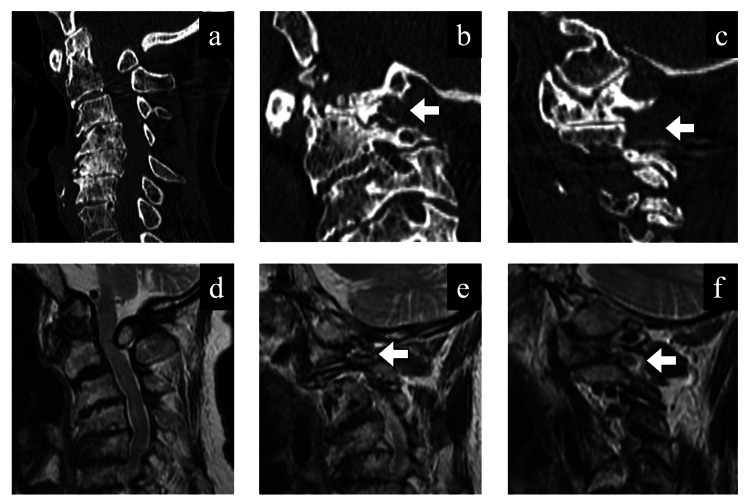
Preoperative computed tomography (CT) and magnetic resonance imaging (MRI) findings. (a, d) Sagittal CT and MRI views showing severe spinal cord compression at the C1 level and mild cervical canal stenosis from C3 to C7. (b, e) Parasagittal CT and MRI views of the right C1–C2 neural foramen demonstrating grade 3 foraminal stenosis due to joint space narrowing and osteophyte formation at the lateral atlantoaxial joint (white arrows). (c, f) Parasagittal views of the left C1–C2 foramen showing no stenosis; white arrows indicate the corresponding foramen for comparison.

CT and MRI revealed severe spinal cord compression at C1 and mild cervical canal stenosis from C3 to C7 (Figure 2A, 2D). Parasagittal CT and MRI demonstrated grade 3 foraminal stenosis, based on Yi’s classification [12], in the right C1-C2 neural foramen (Figure 2B, 2E), due to joint space narrowing and osteophyte formation at the lateral atlantoaxial joint. No stenosis was observed on the left side (Figure 2C, 2F).

**Figure 3:**
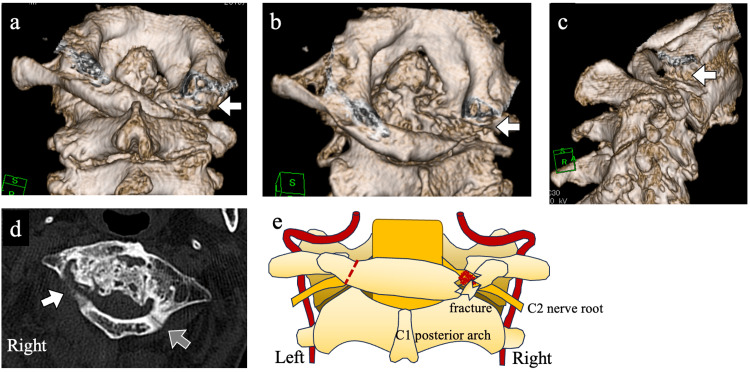
Preoperative computed tomography (CT) findings and schematic illustration based on intraoperative observations. (a–c) Three-dimensional CT views of the cervical spine showing deformities of the C1 posterior arch, initially presumed to result from degenerative change or long-standing rheumatoid arthritis. Intraoperatively, these were identified as fractures (arrows). (d) Axial CT image showing subtle fracture lines in the posterior arch of C1. A fracture line is visible on the left side (gray arrow), while no definitive fracture is seen on the right (white arrow), where the cortical disruption was unclear. (e) Schematic illustration showing fractures of the C1 posterior arch and impingement of the right C2 nerve root by a small bone fragment.

Three-dimensional CT showed significant deformity of the C1 posterior arch (Figure 3A-3C), initially presumed to result from long-standing rheumatoid arthritis or degenerative change. However, intraoperatively, these were identified as unrecognized fractures (Figure 3A-3C, arrows). Retrospective review of axial CT images revealed subtle fracture lines in the posterior arch of C1 (Figure 3D): the right side appeared indistinct (white arrow), while a fracture line was visible on the left (gray arrow). A schematic illustration is shown in Figure 3E.

**Figure 4:**
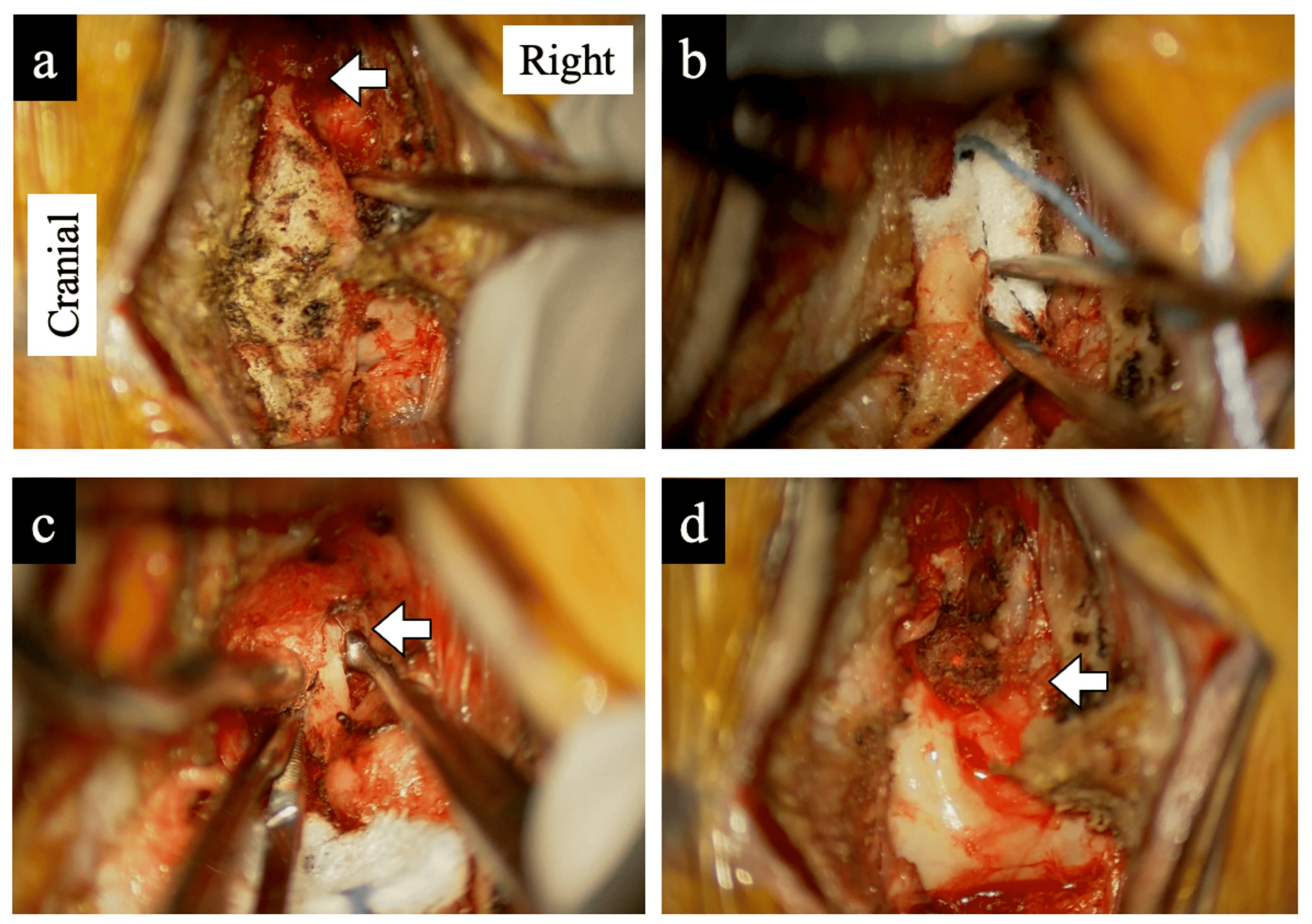
Intraoperative microscopic findings. (a) Exposure of the C1 posterior arch under a surgical microscope. A fracture was clearly visible (arrow), which had not been apparent preoperatively. (b) The right hemi lamina was resected at the fracture site. (c) The right C2 nerve root was found to be compressed by a small bone fragment (arrows), which was removed with a curette. (d) Decompression was achieved, although slight indentation remained on the surface of the C2 nerve root (arrows).

Due to the severity of her symptoms and the lack of sustained improvement, microscopic decompression was performed, consisting of a C1 laminectomy with right-sided foraminotomy at C1-C2 (Video 1). Under general anesthesia, the patient was placed prone, and a midline posterior cervical incision was made. Using an operating microscope, we exposed the C1 lamina (Figure 4A) and identified definitive fractures in the posterior arch (arrow), which had not been apparent preoperatively. The lamina was resected in two steps: the right lamina was widened at the fracture site (Figure 4B), and the left lamina was cut at the lateral margin of the spinal canal. The unstable residual lamina was then removed, exposing the C2 nerve root. The nerve root was compressed by small bone fragments (Figure 4C, arrows), which were removed using a curette. Decompression was achieved (Figure 4D), although slight indentation remained on the surface of the C2 root. A suction drain was placed, and the wound was closed.

**Video 1:** Intraoperative video demonstrating microscopic decompression at the C1–C2 level. The posterior arch of C1 was exposed, and bilateral fractures were identified. The right hemi lamina was resected at the fracture site, followed by resection of the left lamina at the lateral margin. A small bone fragment compressing the right C2 nerve root was removed using a curette, resulting in adequate decompression.

**Figure 5:**
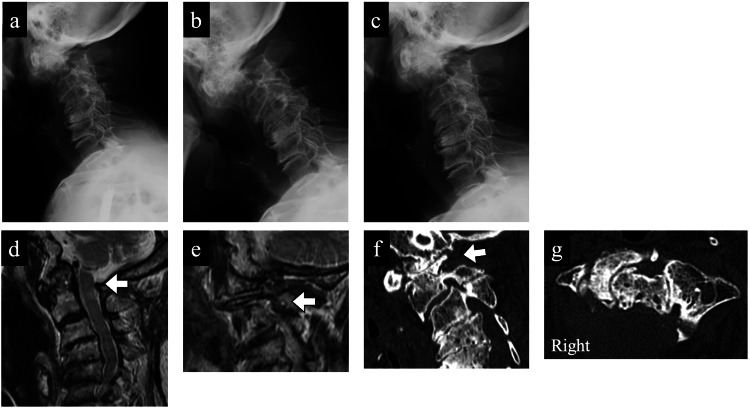
Postoperative radiological findings. (a–c) Dynamic lateral radiographs in flexion, neutral, and extension positions demonstrating no progression of atlantoaxial instability or loss of cervical motion compared to preoperative findings. (d, e) Follow-up magnetic resonance imaging (MRI) confirming sufficient decompression of the spinal cord and right C2 nerve root. (f, g) Postoperative computed tomography (CT) images showing complete removal of the bone fragments within the C1–C2 neural foramen and no evidence of recurrent stenosis or instability.

Immediately after surgery, her VAS pain score decreased to 50 mm. She was ambulatory the following day and was discharged one week later with a soft cervical brace, which she wore for two weeks. At two years postoperatively, her occipital neuralgia had mostly resolved (VAS: 5 mm). Follow-up MRI confirmed sufficient decompression of the spinal cord (Figure 5D) and right C2 nerve root (Figure 5E). CT showed no residual bone fragments in the C1-C2 neural foramen (Figure 5F, 5G), and dynamic radiographs revealed no progression of atlantoaxial instability or loss of cervical motion compared to preoperative findings (Figure 5A-5C).

## Discussion

This case demonstrates C2-related radicular pain associated with a posterior arch defect of C1 in an elderly patient with RA. Although preoperative imaging showed foraminal stenosis, intraoperative observation revealed a small bone fragment ventral to the C2 root contiguous with a posterior arch defect. The distinction between acute fracture and chronic nonunion remains uncertain. Degenerative erosion or congenital cleft cannot be excluded, particularly in osteoporotic bone.

C1-C2 neuroanatomy and pain mechanism

The greater occipital nerve (GON) primarily arises from the medial branch of the dorsal ramus of the C2 spinal nerve, while the lesser occipital nerve (LON) originates from the ventral rami of C2-C3 [13,14]. The C2 dorsal root ganglion (DRG) lies within the foramen between the posterior arch of C1 and the lamina of C2, occupying a large proportion of the foraminal height and making it vulnerable to compression by adjacent osseous or arthritic changes. MRI investigations have demonstrated that narrowing of the C1-C2 neural foramen correlates with irritation of the C2 nerve root and occipital neuralgia [12]. Moreover, bony spurs or fragments from the C1 posterior arch or lateral atlantoaxial joint can impinge on the C2 root and reproduce occipito-cervical pain, which may improve after microscopic decompression [11].

Rheumatoid arthropathy and instability

RA often causes erosive changes in the lateral atlantoaxial joints, leading to instability and foraminal narrowing. Chronic inflammatory bone resorption may also contribute to partial nonunion of the posterior arch. These mechanisms together explain the complex pathology observed here, where the pain origin cannot be attributed solely to a single structure [15-17].

Surgical considerations

Although fusion is often recommended for C1-2 instability, decompression alone was chosen due to advanced age, frailty, and stable preoperative alignment. Microscopic decompression avoided the risks of prolonged anesthesia, instrumentation, and postoperative morbidity. The favorable outcome suggests that limited decompression may be sufficient when instability is minimal.

Educational message

This case underscores the importance of detailed evaluation of the C1-2 foramen when assessing occipital neuralgia. Subtle posterior arch defects may be overlooked on CT, especially in elderly patients with osteoporosis or rheumatoid changes. When conservative therapy fails, targeted decompression can provide meaningful relief without extensive fusion.

Limitations

This is a single case report. The precise nature of the defect (fracture vs nonunion) cannot be confirmed radiologically. Moreover, myelopathic components cannot be fully excluded, and improvement cannot be attributed solely to root decompression. Nonetheless, this case provides valuable insight into the diagnostic process and management of complex C1-2 pathology in elderly patients.

## Conclusions

Posterior arch defects of C1 - whether fracture or nonunion - may cause C2-related radicular pain, particularly in patients with rheumatoid arthritis or atlantoaxial arthropathy. Microscopic decompression without fusion can provide significant and sustained symptom relief in selected elderly patients with stable alignment. Surgeons should carefully assess subtle posterior arch irregularities on CT and MRI when evaluating occipital neuralgia.
